# Sub-classifying patients with mild traumatic brain injury: A clustering approach based on baseline clinical characteristics and 90-day and 180-day outcomes

**DOI:** 10.1371/journal.pone.0198741

**Published:** 2018-07-11

**Authors:** Bing Si, Gina Dumkrieger, Teresa Wu, Ross Zafonte, Alex B. Valadka, David O. Okonkwo, Geoffrey T. Manley, Lujia Wang, David W. Dodick, Todd J. Schwedt, Jing Li

**Affiliations:** 1 School of Computing, Informatics, and Decision Systems Engineering, Arizona State University, Tempe, AZ, United States of America; 2 Department of Neurology, Mayo Clinic, Phoenix, AZ, United States of America; 3 Department of Physical Medicine and Rehabilitation, Harvard Medical School, Spaulding Rehabilitation Hospital, Massachusetts General Hospital, Brigham and Women’s Hospital, Boston, MA, United States of America; 4 Department of Neurosurgery, Virginia Commonwealth University, Richmond, VA, United States of America; 5 Department of Neurological Surgery, University of Pittsburgh, Pittsburgh, PA, United States of America; 6 Department of Neurological Surgery, University of California, San Francisco, CA, United States of America; Julius-Maximilians-Universitat Wurzburg, GERMANY

## Abstract

**Background:**

The current classification of traumatic brain injury (TBI) into “mild”, “moderate”, or “severe” does not adequately account for the patient heterogeneity that still exists within each of these categories. The objective of this study was to identify “sub-groups” of mild TBI (mTBI) patients based on data available at the time of the initial post-TBI patient evaluation and to determine if the sub-grouping correlates with patient outcomes at 90 and 180 days post-TBI.

**Methods:**

Data from patients in the TRACK-TBI Pilot dataset who had a Glasgow Coma Scale (GCS) score of 13 to 15 at arrival to the Emergency Department and a closed head injury were included. Considering 53 clinical variables that are typically available during the initial evaluation of the patient with mild TBI, sparse heirarchial clustering with cluster quality assessment was used to identify the optimal number of patient sub-groups. Patient sub-groups were then compared for ten outcomes measured at 90 or 180 days post-TBI.

**Results:**

Amongst the 485 patients with mTBI, optimal clustering was based on the inclusion of 12 clinical variables that divided the patients into 5 mild TBI sub-groups. Clinical variables driving the sub-clustering included: gender, employment status, marital status, TBI due to falling, brain CT scan result, systolic blood pressure, diastolic blood pressure, administration of IV fluids in the Emergency Department, alcohol use, tobacco use, history of neurologic disease, and history of psychiatric disease. These 5 mild TBI sub-groups differed in their 90 day and 180 day outcomes within several domains including global outcomes, persistence of TBI-related symptoms, and neuropsychological impairment.

**Conclusions:**

Sub-groups of patients with mTBI can be identified according to clinical variables that are relatively easy to obtain at the time of initial patient evaluation. A patient’s sub-group assignment is associated with multidimensional patient outcomes at 90 and 180 days. These findings support the notion that there are clinically meaningful subgroups of patients amongst those currently classified as having mTBI.

## Introduction

The current structure for classifying patients with traumatic brain injury (TBI) includes three main sub-groups defined by Glasgow Coma Scale (GCS) score: mild, moderate, and severe. [1–2] Classifying TBI into these three subgroups has substantial limitations: within each of the three sub-groups there exists a large amount of heterogeneity in patient and injury characteristics and wide variability in post-TBI patient outcomes. For example, a patient who had loss of consciousness for 20 minutes, amnesia for 20 hours, and a GCS score of 13 is categorized as having had a mild TBI (mTBI). Similarly, a patient who had no loss of consciousness, no amnesia, and a GCS score of 15 but had a few minutes of blurred vision and nausea following the head injury is also considered to have had a mTBI. Despite these two patients both being classified as having had “mTBI”, one might expect that the severity and outcomes associated with their injuries may be different. A more refined sub-classification structure for defining the severity of TBI would be useful if the new sub-categories of TBI severity correlated with patient outcomes. Furthermore, a more refined sub-classification of TBI would be most clinically useful if information available at the time of the initial patient evaluation were sufficient for determining an individual’s sub-group. A classification structure with these characteristics could guide patient management decisions and inform appropriate counseling with respect to prognosis.

The objective of this study was to identify sub-classes or “clusters” within a group of patients all currently defined as having mTBI. The sub-classification was determined by considering several heterogeneous characteristics that are typically available when the patient with mTBI presents for initial evaluation and that might relate to patient outcomes: GCS scores, injury characteristics, medical history, substance use, neuroimaging results, vital signs, and basic blood test results. To address the high-dimensionality challenge that is present when using a large number of variables, we used a data-driven approach that automatically selected available clinical features to sub-classify patients and then compared the resulting mTBI sub-groups for their multidimensional 90- and 180-day patient outcomes. In contrast with the majority of existing work that focuses on building predictive models that use clinical variables to predict a single outcome variable of interest, our study has a different perspective in that it allows for the simultaneous consideration of multiple baseline variables and their association with multiple different outcome measures.

## Methods

### Data

The Transforming Research and Clinical Knowledge in Traumatic Brain Injury (TRACK-TBI) Pilot dataset was downloaded from the Federal Interagency Traumatic Brain Injury Research (FITBIR) Informatics System after obtaining appropriate approvals for accessing the data. (tracktbi.ucsf.edu) (fibtir.nih.gov) TRACK-TBI Pilot was conducted between July 2007 and Februray 2011.

The TRACK-TBI Pilot dataset includes multiple Excel files and each file contains different variables of patient characteristics at baseline and patient outcomes at follow-up. A global unique identifier (GUID) is used to track different variables of the same patient across the multiple files. The information used in this study was found in 12 different TRACK-TBI Pilot files, including: Abbreviated Injury Score (AIS), Assessment, CT scan results, injury history, medical history, subject demographics, Glasgow Outcome Score Extended (GOSE) [3], the brief symptom inventory (BSI) [4], the Functional Independence Measure (FIM) [5], the Rivermead Post-Concussion Symptoms Questionnaire (RPQ) [6], the Wechsler Adult Intelligence Scale (WAIS) [7], and the Trail Making Test (TMT) score [8–9]. We used the FITBIR GUID to extract the variables in this study from the FITBIR files and integrate them into one file for all the patients.

In the dataset for this study, each variable was either extracted from one variable in a FITBIR file or was a combination of multiple variables. Specifically, based on domain knowledge we combined the descriptions of injury mechanisms for each patient into 8 categories, including: bike, pedestrian, motorcycle, motor, other person, fall, striking, and other types; IV fluid was a combination of Saline and Crystalloid, i.e. IV fluid = yes if Saline = yes or Crystalloid = yes; vital signs including diastolic and systolic blood pressure, heart rate, and respiratory rate measured at ED arrival and ED discharge were each categorized into three levels, i.e. low, normal, and high, according to clinically-relevant thresholds. O2 saturation was categorized into two levels, i.e. low and normal.

### Patient selection

For these analyses, we selected patients from the TRACK-TBI Pilot dataset who had a GCS score of 13 to 15 at arrival to the Emergency Department and a closed head injury. Of the 599 patients in the dataset, 485 patients met these inclusion criteria.

### Clinical variables

After careful review of the variables available within the TRACK-TBI Pilot dataset, consideration of our own clinical knowledge, and knowledge gained from previously published studies, 53 clinical variables were included as potentially relevant for mTBI sub-classification. [10–19] These variables are listed in Table 1.

**Table 1:** Clinical variables included in analyses.

variable category	variable name	type	values
demographics	age	numerical	years
	gender	binary	0–1: female, male
	ethnicity	binary	0–1: not Hispanic or Latino, Hispanic or Latino
	education	ordinal	1–4: before high, high, college, grad
	employment	binary	0–1: employed, unemployed/not working
	marital status	binary	0–1: married/living together/common law, not married/living together/common law
medical history	alcohol use	binary	0–1: no, yes
	tobacco use	binary	0–1: no, yes
	previous TBI	binary	0–1: no, yes
	prior developmental disease	binary	0–1: no, yes
	prior neurological disease	binary	0–1: no, yes
	prior psychiatric disease	binary	0–1: no, yes
This injury	Intention of injury	binary	0–1: unintentional, intentional
	Injury mechanism—bike	binary	0–1: no, yes
	Injury mechanism—pedestrian	binary	0–1: no, yes
	Injury mechanism—motorcycle	binary	0–1: no, yes
	Injury mechanism—motor	binary	0–1: no, yes
	Injury mechanism—other person	binary	0–1: no, yes
	Injury mechanism—fall	binary	0–1: no, yes
	Injury mechanism—striking	binary	0–1: no, yes
	Injury mechanism—other types	binary	0–1: no, yes
	Injury severity score (ISS)	numerical	0–75
	ISS outside head	binary	0–1: no, yes
ED examination	GCS total score at ED arrival	numerical	13–15
	GCS assessment condition at ED arrival	binary	0–1: no sedation or paralysis, sedation or paralysis
	GCS total score at ED discharge	numerical	3–15
	GCS eye response subscale at ED discharge	numerical	1–4
	GCS motor response subscale at ED discharge	numerical	1–6
	GCS verbal response subscale at ED discharge	numerical	1–5
	GCS assessment condition at ED discharge	binary	0–1: no sedation or paralysis, sedation or paralysis
	hospital type	binary	0–1: primary, secondary
	post-traumatic amnesia duration	ordinal	1–5: none, <1 minute, 1–29 minutes, 30–59 minutes, 1–24 hours
	loss of consciousness duration	ordinal	1–7: none, <1 minute, 1–29 minutes, 30–59 minutes, 1–24 hours, 1–7 days, >7 days
	CT result	binary	0–1: without abnormality, abnormal
	pupil reactivity at ED arrival	ordinal	1–3: both, one, neither reactive
blood work	alcohol intoxication at ED	binary	0–1: no, yes
	any drug intoxication	binary	0–1: no, yes
vital signs	diastolic blood pressure at ED arrival	ordinal	1–3: low (less than 60 mm Hg), normal (60–89 mm Hg), high (at least 90 mm Hg)
	systolic blood pressure at ED arrival	ordinal	1–3: low (less than 90 mm Hg), normal (90–139 mm Hg), high (at least 140 mm Hg)
	heart rate ate ED arrival	ordinal	1–3: low (less than 60 bpm), normal (60–100 bpm), high (at least 101 bpm)
	O2 saturation at ED arrival	ordinal	1–2: low (less than 90%), normal (at least 90%)
	respiratory rate at ED arrival	ordinal	1–3: low (less than 12/min), normal (12-20/min), high (at least 21/min)
	diastolic blood pressure at ED discharge	ordinal	1–3: low (less than 60 mm Hg), normal (60–89 mm Hg), high (at least 90 mm Hg)
	systolic blood pressure at ED discharge	ordinal	1–3: low (less than 90 mm Hg), normal (90–139 mm Hg), high (at least 140 mm Hg)
	heart rate at ED discharge	ordinal	1–3: low (less than 60 bpm), normal (60–100 bpm), high (at least 101 bpm)
	O2 saturation at ED discharge	ordinal	1–2: low (less than 90%), normal (at least 90%)
	respiratory rate at ED discharge	ordinal	1–3: low (less than 12/min), normal (12-20/min), high (at least 21/min)
complications and treatment at ED	hypotension	binary	0–1: no, yes
	seizure	binary	0–1: no, yes
	hypoxia	binary	0–1: no, yes
	IV fluid	binary	0–1: no, yes
	blood transfusion	binary	0–1: no, yes
	intubation	binary	0–1: no, yes

### Outcome variables

We included 10 patient outcome measures: the Glasgow Outcome Score Extended (GOSE) [3] at 90 and 180 days, as well as eight others at 180 days (data on these outcome variables at 90 days were not recorded in the TRACK-TBI Pilot Study) including the brief symptom inventory (BSI) [4], the motor and cognition subscores of the Functional Independence Measure (FIM) [5], the cognition, emotion and somatic subscores of the Rivermead Post-Concussion Symptoms Questionnaire (RPQ) [6], the digit span score from the Wechsler Adult Intelligence Scale (WAIS) [7], and the Trail Making Test (TMT) score [8–9]. These outcome measures evaluate post-TBI global outcomes, psychological status, TBI related symptoms, physical function, and cognitive activity limitations/ neuropsychological impairment. Literature search and domain knowledge were used to binarize each outcome score into “good” and “bad” outcomes. Table 2 shows the outcome variables and rules for binarization. [20–26]

**Table 2:** Outcome variables and rules for binarization into “good” vs “bad” recovery.

Outcome variables (total and subscales)	Binarization	Availability at 90 days	Availability at 180 days
Glasgow Outcome Scale Extended (GOSE) [26]	1–6 (bad outcome) 7–8 (good outcome)	yes	yes
Brief System Inventory (BSI) [20]	GSI T-score > 63 or two or more subscales with T-score > 63 (bad outcome); otherwise (good outcome)	no	yes
Functional Independence Measure (FIM)–Motor [21]	all responses 6 or higher (good) vs. any response 5 or lower (bad)	no	yes
Functional Independence Measure (FIM)—Cognition [21]	all responses 6 or higher (good) vs. any response 5 or lower (bad)	no	yes
Rivermead Post-Concussion Symptoms Questionnaire (RPQ) -Cognition [22]	any item rated 3 or 4 (bad) vs. no item rated 3 or 4 (good)	no	yes
Rivermead Post-Concussion Symptoms Questionnaire (RPQ)–Emotion [22]	any item rated 3 or 4 (bad) vs. no item rated 3 or 4 (good)	no	yes
Rivermead Post-Concussion Symptoms Questionnaire (RPQ)–Somatic [22]	any item rated 3 or 4 (bad) vs. no item rated 3 or 4 (good)	no	yes
Wechsler Adult Intelligence Scale 4^th^ edition (WAIS) Processing Speed Index [25]	> = 1 standard deviation below the mean (bad)	no	yes
Trail Making Test (TMT) [23–24]	Age adjusted normalized times. Bad = 1 standard deviation above mean or more. A "Bad" outcome overall would be the result of a "Bad" outcome on A or B individually.	no	yes

### Clinical data imputation

Because the sub-classification was performed on clinical variables, we only imputed missing data for clinical variables, but did not impute data for missing outcome variables. We only included clinical variables for which fewer than 10% of patients had missing values, except for several TBI-related variables that we hypothesized to be exceptionally important for sub-classifying patients: post-traumatic amnesia (PTA) duration, loss of consciousness (LOC) duration, previous TBI, and pupil reaction at ED arrival. As a classic imputation method, Multivariate Imputation by Chained Equations (MICE) implemented in the R package “mice” [27] was used to impute missing data. MICE works by building a series of regressions with missing data conditional upon observed data. Logistic regression is used if the variable with missing data is binary; multinomial logit regression is used if the variable is categorical with more than two levels; linear regression is used if the variable with missing data is numerical. The quality of imputation was confirmed by comparing the empirical distributions of each variable before and after the imputation and finding no statistically significant difference between the distributions.

### Sparse hierarchical clustering (SHC) with cluster quality assessment

Hierarchical Clustering (HC) is a conventional clustering algorithm to build a hierarchy of subgroups by producing a dendrogram that represents a nested set of subgroups. HC starts from the bottom of the dendrogram where each subject is in its own subgroup and the pairwise distance between the subgroups is measured. In the next upper level of the dendrogram, the pair of subgroups with the closest distance is merged into a bigger subgroup and the dendrogram is thus iteratively built. However, when the number of variables used in HC is large, like in this study, Sparse Hierarchical Clustering (SHC) [28] is more appropriate than conventional HC. SHC can automatically select informative features to the clustering result, or in other words, automatically eliminate features that do not contribute to sub-classifying patients.

Conventional HC is based on an overall distance matrix between each pair of samples, i.e. **U**. Instead of **U**, SHC builds a dendrogram based on a weighted distance matrix **D**, where each clinical variable is associated with a weight. An L1-penalty [29] is imposed on the weights to make sure the estimated weights are “sparse”, i.e., to shrink the estimated weights for clinical variables not significantly contributing to the clustering to be exactly zero. Mathematically, SHC solves the following optimization problem to estimate the weight vector **w** and the weighted distance matrix **D**:

$$\begin{array}{c} (w^{*},D^{*})=\underset{w,D}{\mathrm{argmax}}\left\{\sum_{j}w_{j}\sum_{i,i^{\prime }}u_{i,i^{\prime },j}D_{i,i^{\prime }}\right\}\\ subject\;to\;\sum_{i,i^{\prime }}D_{i,i^{\prime }}^{2}\leq 1,\;{\left\|w\right\|}_{2}\leq 1,\;{\left\|w\right\|}_{1}\leq s,\;w_{j}\geq 0,\;for\;j=1,\ldots ,q. \end{array}$$ (1)

Here, *u*_*i*,*i*′,*j*_ is the distance between samples *i* and *i*′ on the *j*-th clinical variable. *w*_*j*_ is the *j*-th element in the weight vector ***w***, i.e., the weight for the *j*-th clinical variable. *q* is the number of clinical variables. ‖**w**‖_1_ is an L1-penalty defined as a summation of the absolute weights. The constraint on ‖**w**‖_1_ is to impose sparsity. ‖**w**‖_2_ is the L2-norm, which is used together with other constraints in (1) to facilitate efficiency of the optimization solution. **D**_*i*,*i*′_ is an element in **D** representing the weighted distance between samples *i* and *i*′. *s* is a tuning parameter. Different values for the tuning parameter (*s*) result in different numbers of clinical variables with non-zero weights. To determine the optimal number of clinical variables, a gap statistic is computed, which measures the strength of the clustering obtained on the real data relative to the clustering obtained on permuted data that does not contain subgroups [28]. The optimal number of clinical variables is the one that maximizes the gap statistic. SHC has been implemented in the R package “sparcl”, which was used to perform the analysis in this study.

Furthermore, depending on where the dendrogram is cut, SHC can produce different numbers of subgroups. To determine the optimal number of subgroups, we adopted a cluster quality measure proposed by Kapp and Tibshirani [30] called “in group proportion (IGP)”. IGP is defined to be the proportion of samples classified to a cluster whose nearest neighbor is also classified to the same cluster. According to this definition, IGP is between 0 and 1; and the higher the IGP, the better quality of the cluster. Mathematically, IGP for a cluster *c* is defined as:

$$IGP(c)=\frac{\#\{i|Class(i)=Class(i^{N})=c\}}{\#\{i|Class(i)=c\}},$$ (2)

where *Class* (*i*) is the cluster membership of sample *i*; *i*^*N*^ is *i*’s nearest neighbor who can be found from the estimated weighted distance matrix D^*^ using SHC. We determined the cut of the dendrogram (i.e., the optimal number of subgroups) that produced a high overall IGP across all the resulting clusters.

### Sparse outcome selection (SOS)

After the subgroups are found, they are compared with each other in terms of multi-dimensional outcome variables. For a pair of subgroups, e.g., *c*_0_ and *c*_1_, the goal is to find the subset of outcome variables on which *c*_0_ and *c*_1_, significantly differ. Conventional methods would perform a hypothesis testing on each outcome variable and use FDR to control the overall type I error. However, FDR is known to be too conservative. [31] Thus, sparse learning was used to identify the subset of outcome variables simultaneously, which overcomes the weakness of multiple comparisons and FDR. Specifically, suppose there are *p* outcome variables. Let *y*_*i*_ = 1 or 0 representing bad or good recovery for the *i*-th outcome variable, *i* = 1,…,*p*. Let *x* = 1 or 0 representing subgroups *c*_1_ and *c*_0_. A logistic regression model can be used to link the subgroup membership of each patient with the log-odds of bad recovery in terms of the *i*-th outcome variable, i.e.,

$$\log\frac{P(y_{i}=1)}{P(y_{i}=0)}=\alpha_{i}+\beta_{i}x,$$ (3)

where *α*_*i*_ is the log-odds of bad recovery for subgroup *c*_0_ and *β*_*i*_ represents the increase of log-odds for subgroup *c*_1_ compared with *c*_0_. If *β*_*i*_ = 0, it means that the two subgroups do not differ on the *i*-th outcome. To simultaneously identify the subset of outcomes on which the two subgroups differ, we imposed an L1-penalty on the joint log-likelihood function over all the outcomes, i.e.,

$$(\alpha^{*},\beta^{*})={\mathrm{argmin}}_{\alpha ,\beta }-\sum_{j=1}^{p}l_{j}(\alpha_{j},\beta_{j})+\lambda_{1}{\left\|\beta \right\|}_{1},$$ (4)

where *l*_*j*_(*α*_*j*_,*β*_*j*_) is the log-likelihood function corresponding to the *i*-th outcome based on the model in (3). **β** = (*β*_1_,…,*β*_*p*_) and **α** = (*α*_1_,…,*α*_*p*_). The L1-penalty on **β**, i.e., ‖**β**‖_1_, has the effect of shrinking the *β*_*i*_’s with a small magnitude to be exactly zero and the remaining non-zero *β*_*i*_’s compose the subset of outcomes on which the two subgroups significantly differ. *λ*_1_ is a tuning parameter that can be selected by minimizing the cross-validated deviance.

## Results

In this section, we first introduce the summary statistics to describe the clinical variables from TRACK-TBI Pilot dataset used in our study. Then, we present sub-classification based on clinical variables, followed by characterization of each subgroup with clinical variables and multi-dimensional outcomes. To compare clinical variables and outcomes between each pair of clusters, a Chi-square test for proportions is used, from which a p-value can be provided to indicate the significance of the test result.

Summary statistics for the variables included in this analysis and the percentage of missing data for each variable are shown in Table 3. Variables that significantly contributed to patient sub-classification are indicated with “*”.

**Table 3:** Summary statistics of clinical variables included in the analysis.

variable category	variable name	summary statistics (frequency/mean)	summary statistics (%)	percentage of data missing
demographics	Age (mean +/- SD)	42.6 +/- 18.7		0%
	*gender (female/male)	138/347	28.5/71.5	0%
	Ethnicity (not Hispanic or Latino/Hispanic or Latino)	410/75	84.5/15.5	0.4%
	Education (before high/ high/college/grad)	89/246/102/48	18.4/50.7/21/ 9.9	5.8%
	*Employment (employed/unemployed)	283/202	58.4/41.6	6.8%
	*marital status (married/not married)	156/329	32.2/67.8	5.4%
medical history	*alcohol use (no/yes)	240/245	49.5/50.5	0%
	*tobacco use (no/yes)	326/159	67.2/32.8	0%
	previous TBI (no/yes)	134/351	27.6/72.4	39.8%
	prior developmental disease (no/yes)	431/54	88.9/11.1	0%
	*prior neurological disease (no/yes)	292/193	60.2/39.8	0%
	*prior psychiatric disease (no/yes)	336/149	69.3/30.7	0%
this injury	Intention of injury (unintentional/intentional)	418/67	86.2/13.8	3.5%
	Injury mechanism–bike (no/yes)	404/81	83.3/16.7	0%
	Injury mechanism–pedestrian (no/yes)	450/35	92.8/3.2	0%
	Injury mechanism–motorcycle (no/yes)	472/13	97.3/26.8	0%
	Injury mechanism–motor (no/yes)	395/90	81.4/18.6	0%
	Injury mechanism—other person (no/yes)	405/80	83.5/16.5	0%
	*Injury mechanism–fall (no/yes)	329/156	67.8/32.2	0%
	Injury mechanism–striking (no/yes)	469/16	96.7/3.3	0%
	Injury mechanism—other types (no/yes)	471/14	97.1/2.9	0%
	Injury severity score (ISS) (mean +/- SD)	9.4 +/- 9.7		0%
	ISS outside head (no/yes)	107/378	22.1/77.9	7.2%
ED examination	GCS total score at ED arrival (mean +/- SD)	14.7 +/- 0.5		0%
	GCS assessment condition at ED arrival (no sedation or paralysis/sedation or paralysis)	480/5	99/1	0.6%
	GCS total score at ED discharge (mean +/- SD)	14.4 +/- 2.2		6.6%
	GCS eye response subscale at ED discharge (mean +/- SD)	3.8 +/- 0.6		6.0%
	GCS motor response subscale at ED discharge (mean +/- SD)	5.8 +/- 0.9		6.2%
	GCS verbal response subscale at ED discharge (mean +/- SD)	4.7 +/- 0.8		6.6%
	GCS assessment condition at ED discharge (no sedation or paralysis/sedation or paralysis)	392/93	80.8/17.2	5.6%
	hospital type (primary/secondary)	402/83	83/17	0.6%
	post-traumatic amnesia duration (none/<1 minute/1-29 minutes/30-59 minutes/1-24 hours)	202/35/124/47/ 77	41.6/7.2/25.6/ 9.7/15.9	19.2%
	loss of consciousness duration (none/<1 minute/1-29 minutes/30-59 minutes/1-24 hours/1-7 days/>7 days)	134/71/211/36/ 26/3/4	27.6/14.6/43.5/ 7.4/5.4/0.6/0.8	22.3%
	*CT result (without/with abnormality)	255/230	52.6/47.4	2.5%
	pupil reactivity at ED arrival (both/one/neither reactive)	476/6/3	98.1/1.2/0.6	15.9%
blood work	alcohol intoxication at ED (no/yes)	458/27	94.4/5.6	0%
	any drug (no/yes)	458/27	94.4/5.6	0%
vital signs	*diastolic blood pressure at ED arrival (low/normal/high)	0/329/156	0/67.8/32.2	19.6%
	*systolic blood pressure at ED arrival (low/normal/high)	0/257/228	0/53.0/47.0	2.1%
	heart rate ate ED arrival (low/normal/high)	26/346/113	5.4/71.3/23.3	0.8%
	O2 saturation at ED arrival (low/normal)	2/483	0.4/99.6	3.9%
	respiratory rate at ED arrival (low/normal/high)	6/427/52	1.2/88/10.7	2.3%
	diastolic blood pressure at ED discharge (low/normal/high)	0/434/51	0/89.5/10.5	19.8%
	systolic blood pressure at ED discharge (low/normal/high)	0/365/120	0/75.3/24.7	5.2%
	heart rate ate ED discharge (low/normal/high)	16/425/44	3.3/87.6/9.1	5.0%
	O2 saturation at ED discharge (low/normal)	1/484	0.2/99.8	9.3%
	respiratory rate at ED discharge (low/normal/high)	5/466/14	1.0/96.1/2.9	6.6%
complications and treatment	Hypotension (no/yes)	475/10	97.9/2.1	0.6%
	Seizure (no/yes)	475/10	97.9/2.1	0.6%
	Hypoxia (no/yes)	463/22	95.5/4.5	0.6%
	*IV fluid (no/yes)	192/293	39.6/60.4	0%
	blood transfusion (no/yes)	473/12	97.5/2.5	0%
	Intubation (no/yes)	470/15	97.9/2.1	0%

Clinical variables found by sparse hierarchical clustering that significantly contribute to sub-classifying TRACK-TBI Pilot patients are indicated by “*”. SD = Standard Deviation.

### Sub-classification based on clinical variables

**Fig 1:**
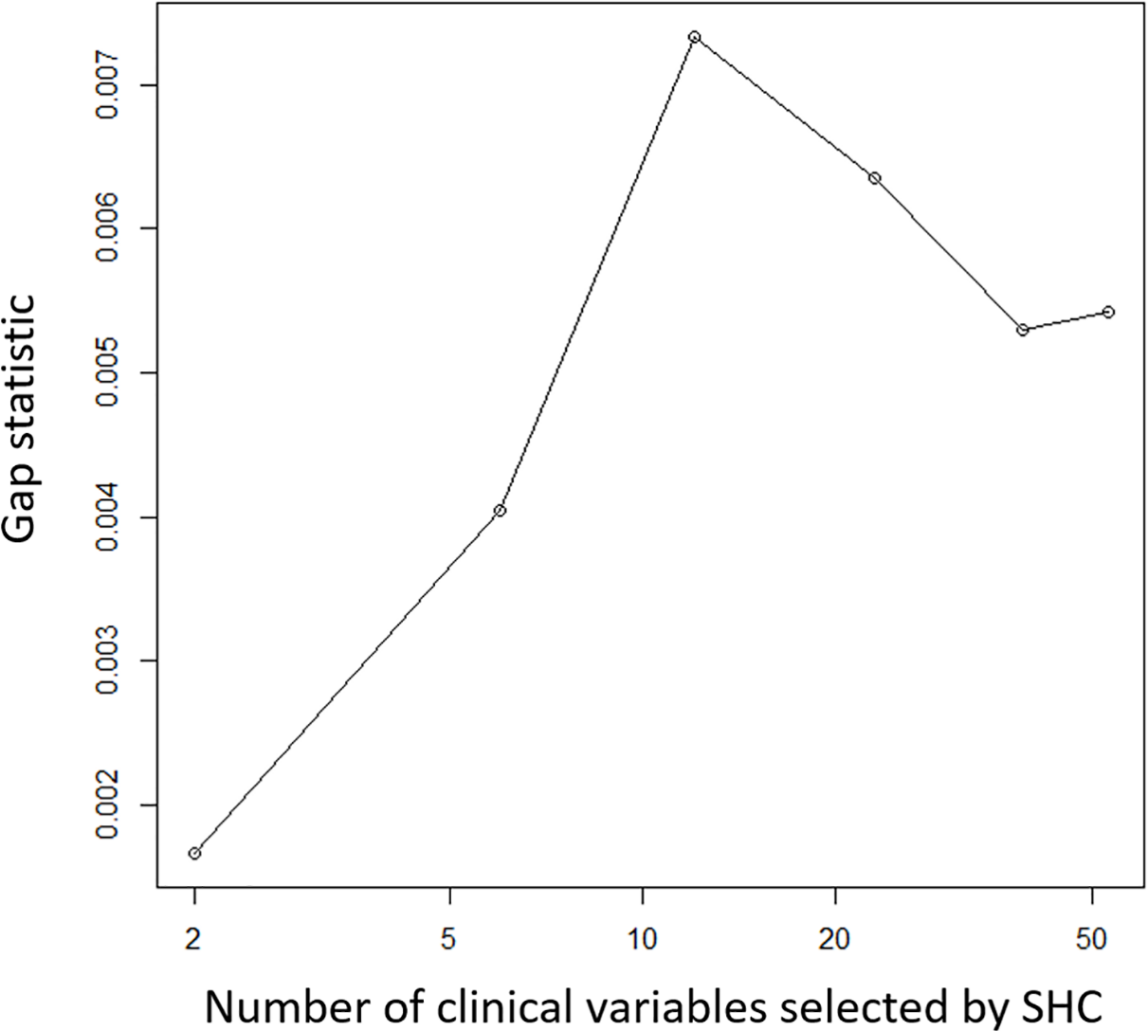
Based on the gap statistic it was determined that 12 clinical variables provided optimal clustering of patients with TBI.

**Fig 2:**
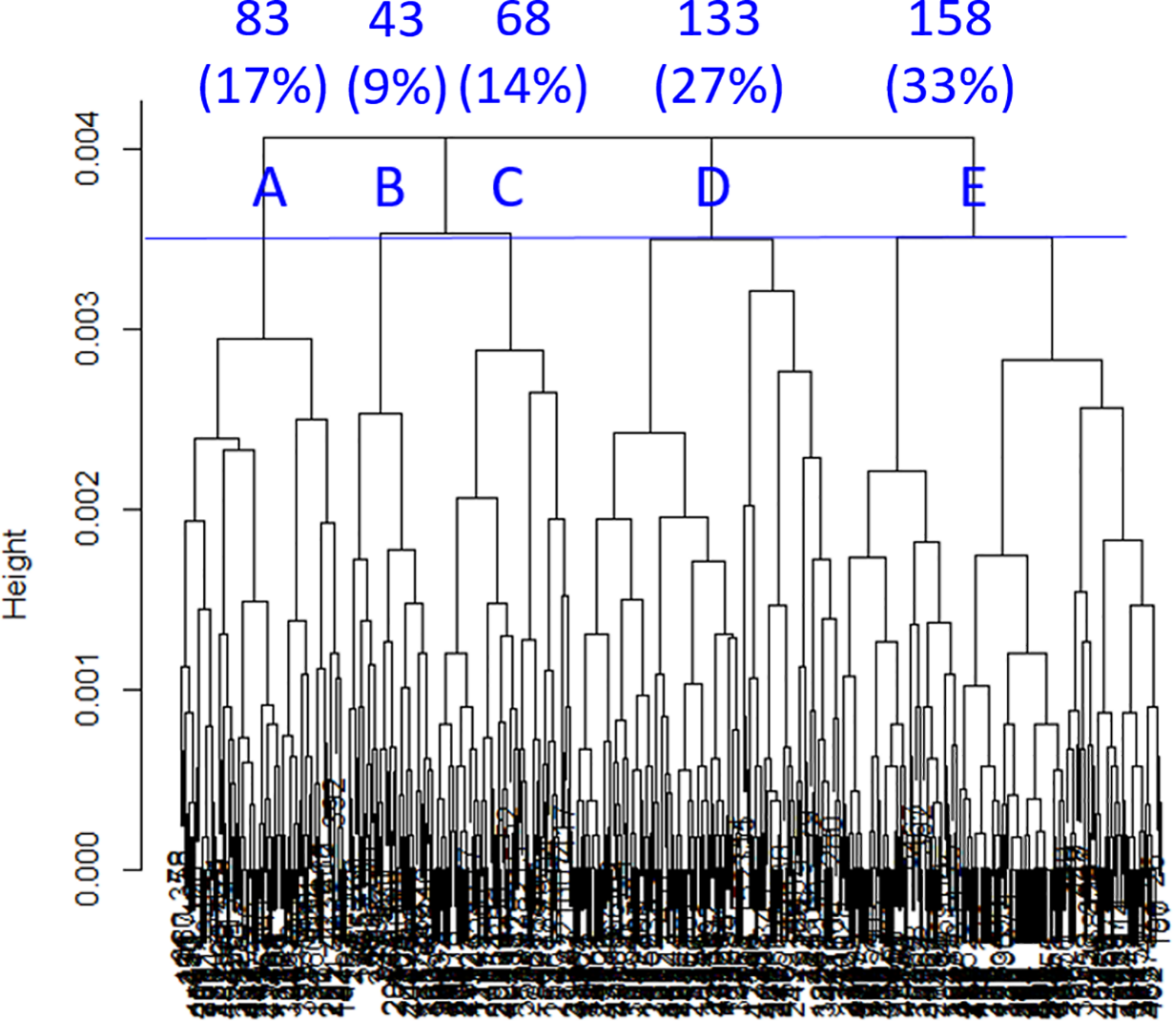
Dendrogram built by sparse hierarchical clustering showing the five clusters (A-E) of patients with TBI used in subsequent analyses. The percentages refer to the proportion of TRACK-TBI Pilot patients assigned to each cluster.

Inclusion of 12 clinical variables maximized the gap statistic in SHC (Fig 1). These variables are highlighted using “*” in Table 3. Using the 12 clinical variables, SHC produced the dendrogram shown in Fig 2. The optimal cut was found using IGP, which produced five clusters (i.e. subgroups of patients with TBI), whose IGPs are 0.95, 1.00, 0.98, 0.98, and 0.99 for clusters A-E, respectively. Clusters A-E included 17%, 9%, 14%, 27%, and 33% of all the TRACK-TBI Pilot subjects respectively.

### Subgroup characterization using clinical variables

**Fig 3:**
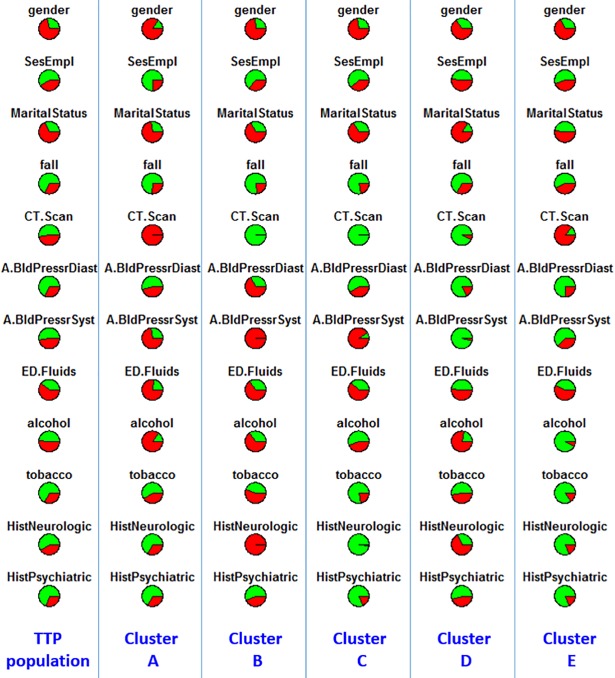
Distributions of clinical variables in clusters A-E and in entire TRACK-TBI Pilot population. Red represents proportions of males, unemployment, unmarried, injury due to falls, abnormal CT, abnormal diastolic blood pressure at ED arrival, abnormal systolic blood pressure at ED arrival, use of fluid at ED, use of alcohol, use of tobacco, history of neurologic diseases, and history of psychiatric diseases. SesEmpl = employment; CT.Scan = CT result; A.BldPressrDiast = diastolic blood pressure at ED arrival; A.BldPressrSyst = systolic blood pressure at ED arrival; ED.Fluids = IV fluid; alcohol = alcohol use; tobacco = tobacco use; HistNeurologic = prior neurological disease; HistPsychiatric = prior psychiatric disease; TTP = TRACK-TBI Pilot.

We used a “pie chart array” to visualize the distributions of the 12 clinical variables within each cluster, as shown in Fig 3. Each pie in the column corresponds to one of the 12 clinical variables in Table 3 that significantly contributed to the clustering. Red and green colors of each pie represent the proportions of the two levels for each variable (all 12 variables are binary).

The most obvious difference between the five clusters is the proportion of patients with brain CT abnormality. Clearly, almost all the patients with an abnormal CT (i.e. “complicated mTBI”) fall into clusters A and E, while almost all the patients with a normal CT fall into cluster B, C, and D. Table 4 shows the percentage of patients with an abnormal CT in each cluster.

**Table 4:** Percentage of patients with an abnormal brain CT in each cluster.

	A	B	C	D	E
% patients with an abnormal CT	100%	0%	0%	7.5%	86.7%

Please note although B and C are the same in terms of both having 100% patients with a normal CT, they differ significantly in some other variables. Specifically, B has higher percentages of tobacco use (p<0.001), neurologic disease history (p<0.001), and psychiatric disease history (p = 0.004) than C.

Similarly, we compare D vs B, and D vs C. D is similar to B and C in terms of having 92.5% patients with a normal CT. However, D has lower percentages of abnormal diastolic & systolic blood pressure at ED (p<0.001, p<0.001) and neurologic disease history (p<0.001), compared with B. If comparing with C, D has higher percentages of alcohol use (p<0.001), tobacco use (p<0.001), neurologic disease history (p<0.001), and psychiatric disease history (p<0.001), a higher percentage of unmarried people, and lower percentages of abnormal diastolic & systolic blood pressure at ED (p<0.001, p<0.001).

Furthermore, although A and E are similar in terms of CT scans, A has a lower percentage of unemployment (p = 0.001), a higher percentage of unmarried people (p = 0.001), and higher percentages of abnormal diastolic & systolic blood pressure at ED (p = 0.001, p<0.001), fluid use (p = 0.002), and alcohol and tobacco use (p<0.001, p< 0.001).

### Multi-dimensional outcomes of subgroups

**Fig 4:**
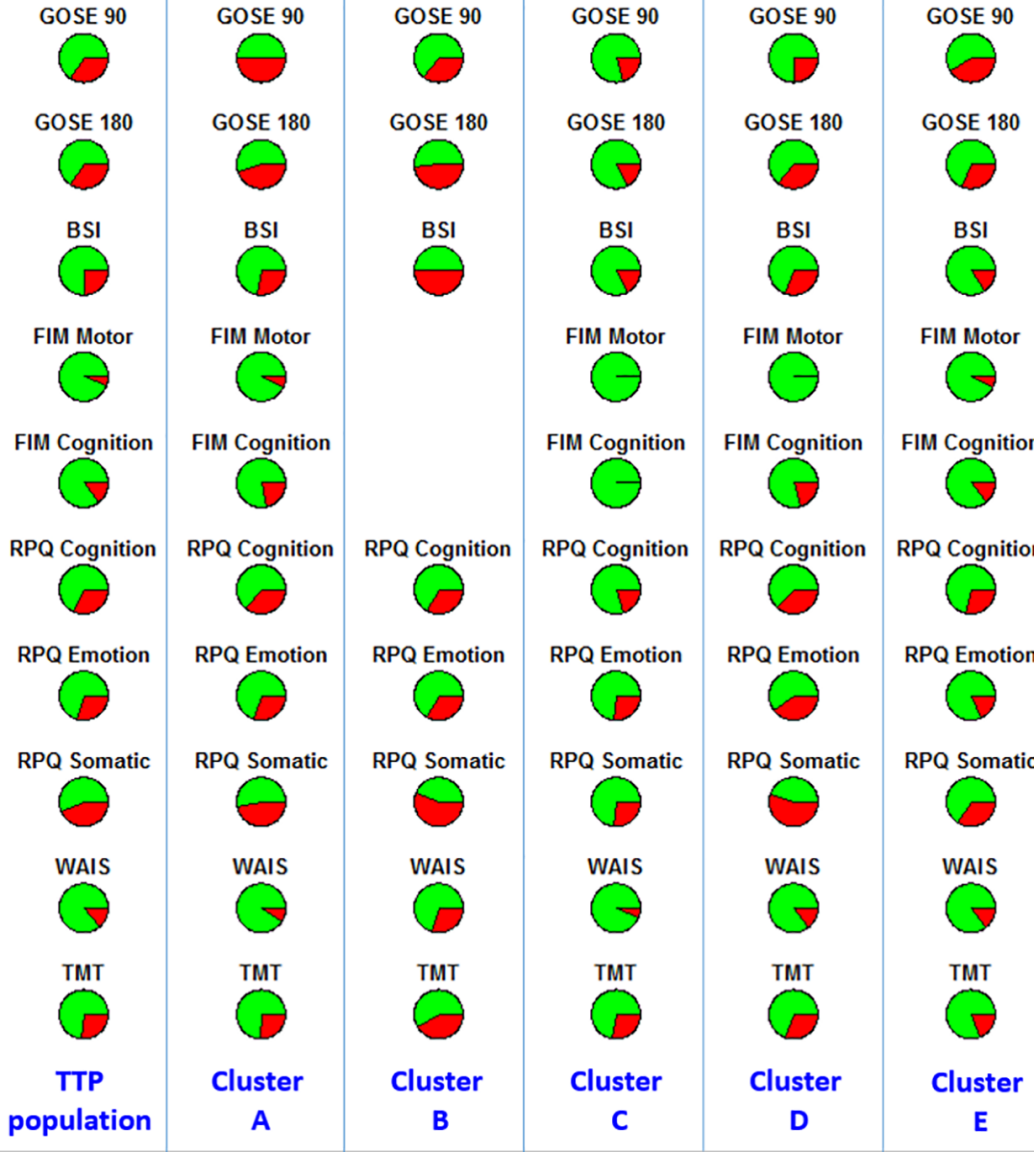
Distributions of “good” (green) and “bad” (red) outcomes in the entire TRACK-TBI Pilot population and in each cluster. (No patient in cluster B had FIM Motor and Cognition measurements, so they are left blank). GOSE 90 = GOSE at 90 days; GOSE 180 = GOSE at 180 days; BSI = BSI at 180 days; FIM Motor = FIM–Motor at 180 days; RPQ Cognition = RPQ–Cognition at 180 days; RPQ Emotion = RPQ–Emotion at 180 days; RPQ Somatic = RPQ–Somatic at 180 days; WAIS = WASI at 180 day; TMT at 180 days.

Table 5 shows the proportion of patients in the entire TRACK-TBI Pilot population with “bad” post-TBI outcomes for GOSE, BSI, FIM motor and cognitive subscales, RPQ cognitive, emotional and somatic outcomes, WAIS, and TMT. The definitions of “bad” outcomes are in Table 2. We used a “pie chart array” to help visualize the distributions of outcome variables within each cluster, as shown in Fig 4.

**Table 5:** Proportion of patients with poor recovery in the entire TRACK-TBI Pilot population.

	GOSE 90 days	GOSE 180 days	BSI	FIM Motor	FIM Cognition	RPQ Cognition	RPQ Emotion	RPQ Somatic	WAIS	TMT
Number of Patients	362	318	277	80	80	274	274	274	247	248
Proportion with bad outcomes	0.345	0.344	0.249	0.063	0.150	0.318	0.292	0.434	0.138	0.266

The SOS approach was used to compare outcomes in sub-groups B, C and D (the “normal brain CT” groups). It was found that B and C differ on GOSE180, BSI, RPQ Somatic, and WAIS. Chi-square tests for proportions on these four outcome measures confirmed that B has significantly worse recovery across the four outcome measures (p = 0.01, 0.01, 0.04, 0.05). We contend that this is because although all the patients in B and C have a normal CT, B is worse than C in terms of more tobacco use and neurologic/psychiatric disease history. This seems to affect the recovery on specific domains like BSI, RPQ somatic, and WAIS, and interestingly also the overall recovery measured by GOSE at 180 days. Comparing the outcome measures in D and C, SOS found that D and C differ on GOSE180 and RPQ Somatic. Chi-square tests for proportions on these two outcome measures confirmed that D has significantly worse recovery across the two outcome measures (p = 0.03, 0.005). Recall that D has higher percentages of alcohol use (p<0.001), tobacco use (p<0.001), neurologic disease history (p<0.001), and psychiatric disease history (p<0.001), which seem to affect the recovery on both RPQ somatic and GOSE 180. This is in line with the previous finding on B and C. SOS did not find any outcome measure that significantly differs between D and B, implying that patient difference observed at the time of their clinical assessment did not lead to outcome differences.

Comparing the outcome measures in A and E, SOS found that A and E differ only on GOSE180, with A having significantly worse overall recovery (p = 0.05). After removing the 13.3% patients with a normal CT from E, we applied SOS again on A and E_sub (i.e., the subset of patients in E with an abnormal CT). SOS, again, only found GOSE 180 differed between groups, but with an even smaller p value of 0.04. This indicates that for two clusters with 100% abnormal CT (i.e., A and E_sub), patients’ overall recovery tends to be worse for the cluster whose patients are “unhealthy” in terms of other metrics such as alcohol and tobacco use, diastolic & systolic blood pressure, and use of IV fluids in the ED (i.e., cluster A). Also, A has a significantly lower percentage of unemployment and a higher percentage of unmarried people. This implies demographics may also play a role in overall recovery.

Finally, we compared the outcome measures between the CT normal vs abnormal patients. This is similar to combining A and E into one cluster and B, C, D into another cluster. Since we know that A and E have substantial heterogeneity in terms of clinical variables other than CT (so do B, C, and D), we wanted to interrogate what would happen if we ignored this heterogeneity. Interestingly, GOSE score measured at 90 days post-TBI (but not GOSE score measured at 180 days post-TBI) was found to be significantly different between the CT normal and abnormal groups, with the CT normal group having a significantly better recovery than the CT abnormal group (p<0.001). Note that within the 10 outcomes we focused on in this paper, only GOSE was measured at 90 and 180 days, while others were only measured at 180 days. This means that CT abnormality seems to have a more substantial impact on near-term outcomes (i.e., at 90 days), while delineation of the heterogeneity beyond CT helps predict longer-term outcomes and on more dimensions.

## Discussion

The main objective of this study was to identify sub-groups of patients within a population of individuals who presented with mild TBI (GCS scores of 13–15). Identifying sub-groups based upon clinical data that are typically available at the time a patient with TBI is initially evaluated could allow for recognition of baseline clinical variables that associate with “good” or “bad” patient outcomes. The main finding of this study is that 5 sub-groups of patients were identified within the larger patient population. The twelve clinical variables that contributed to the clustering structure included: gender, employment status, marital status, injury mechanism, head CT findings, systolic blood pressure, diastolic blood pressure, receiving IV fluids while in the ED, having a history of alcohol use, a history of tobacco use, a history of psychiatric disease, and a history of neurologic disease. Patients in each of the five clusters have different outcomes in regards to global post-TBI outcomes (e.g. GOSE), psychological health (e.g. BSI), cognition (e.g. WAIS), and post-TBI related symptoms (RPQ). This study helps to identify patient variables that should be further investigated when developing and validating prognostic models for TBI and when identifying more precise sub-categories of mTBI that correlate with patient outcomes. Predictive outcome models consisting of data that are easily and routinely collected during the initial evaluation of patients with mTBI would assist the clinician with determining how aggressively to manage the patient and with providing prognoses to the patients.

There are several published studies that have performed univariate analysis on TRACK-TBI Pilot data that utilize clinical characteristics as predictors for patient outcomes. Connor et al. [11] studied the influence of a previous history of TBI with loss of consciousness (LOC) on current TBI and found patients with previous TBI have less-severe acute injuries, but experienced worse outcomes at 180 days, i.e. higher BSI, lower satisfaction with life, lower WAIS-IV, and lower CVLT-II. Yuh et al. [10] investigated univariate predictors of GOSE, including demographics, DTI, MRI, and CT imaging, and clinical characteristics, and discovered MRI evidence for contusion, ≥ 1 ROI with severely reduced fractional anisotropy (FA), neuropsychiatric history, age, and years of education as significant predictors of GOSE at 90 days, and ≥ 1 ROI with severely reduced FA, neuropsychiatric history, and years of education as significant predictors of GOSE at 180 days. Diaz-Arrastia et al. [12] studied two TBI-related biomarkers, ubiquitin C-terminal hydrolase L1 (UCH-L1) and glial fibrillary acidic protein (GFAP), and found no significant association with GOSE at 180 days. Korley et al. [13] investigated the diagnostic and prognostic values of serum brain-derived neurotrophic factor (BDNF), glial fibrillary acidic protein (GFAP), and ubiquitin C-terminal hydrolase-L1 (UCH-L1) by correlating them with the outcomes of RPQ and GOSE at 180 days, and found that the 180-day recovery can be predicted by BDNF with 65% accuracy and the addition of GFAP/UCH-L1 did not significantly improve the prediction. Yue et al. [14] discovered a negative association of the ANKK1 T allele with cognitive outcome measured by the WAIS at 180 days, i.e. T/T patients performed significantly worse than C/T and C/C patients. Winkler et. al. [15] discovered that a single-nucleotide polymorphism (SNP) in catechol-o-methyltransferase (COMT) Met^158^ allele is associated with a lower incidence of PTSD and higher GOSE score at 180 days. A rs6277 T-allele in the dopamine D2 receptor was also found to be associated with better verbal learning and recall on California Verbal Learning Test (CVLT-II) but not with non-verbal processing speed (WAIS-PSI) or cognitive activity measured by the TMT. [13]

Furthermore, there are several studies aiming to find multivariate predictors in order to explore the joint value of multiple predictors on outcomes. Lingsma et al. [17] studied an mTBI subgroup with available 90- or 180-day GOSE scores from TRACK-TBI Pilot and found that lower GOSE scores at 90 and 180 days share the same significant predictors, including older age, pre-existing psychiatric conditions, lower education, injury caused by assault, and extracranial injury in addition to the head trauma. Yuh et al. [18] also performed multivariate modeling to identify factors associated with 90 day patient outcomes, focusing only on a mTBI subgroup that was evaluated with head CT for their mTBI. CT evidence of subarachnoid hemorrhage, unemployment, one or more brain contusions on MRI, and ≥ 4 foci of hemorrhagic axonal injury on MRI were found to be significant predictors of GOSE scores at 90 days. Cnossen et al. [19] developed a predictive model for 180-day post-concussive symptoms for mTBI patients using the least absolute shrinkage and selection operator (LASSO) model and discovered years of education, history of psychiatric disorder, and previous TBI were the strongest predictors for RPQ at 180 days.

Consistent with the previous studies, in our study, CT results, employment status, neurologic disease history and psychiatric disease history were also identified as significant clinical variables contributing to TRACK-TBI Pilot patient sub-classification. Additional factors contributing to sub-classification in our study included: gender, marital status, TBI due to falling, post-TBI blood pressure, use of IV fluids in the emergency department, alcohol use and tobacco use. Furthermore, all of the aforementioned studies performed “supervised” learning of the data, i.e., they assessed how well each clinical variable or combinations of variables can predict an outcome of interest. Our study, on the other hand, performed “unsupervised” learning, i.e., we aimed to find natural subgroups of TBI patients who are relatively homogenous in clinical characteristics within each subgroup and have greater contrast between subgroups. The subgroup membership was then correlated with multidimensional outcome variables. This unsupervised learning approach has advantages over supervised learning in that: 1) The method naturally facilitates multi-domain outcome assessments (physical, psychological, cognitive) of each patient subgroup, and thus eventually could help to determine overall management strategies for patients with mTBI instead of just treating one particular outcome. 2) This method can facilitate genetic and biological discovery of the underlying basis leading to within-subgroup homogeneity and between-subgroup heterogeneity.

There are several limitations of this study including: 1) We did not have access to specific brain CT results, beyond “normal” vs. “abnormal”. It is possible that the type and severity of brain CT abnormality would contribute to mTBI sub-classification and it is likely that these more specific CT data would correlate with patient outcomes. 2) We excluded clinical variables for which more than 10% of patients had missing values, except for several TBI-related variables that we hypothesized to be exceptionally important for sub-classifying patients. It is likely that there are other clinical variables contributing to mTBI sub-classification not included in our analysis. 3) GOSE scores of 7 (lower good recovery) and 8 (upper good recovery) were combined into one group for analyses. A recent publication provides evidence that patients within each of these two categories might have substantially different multidimensional outcomes. [32] Future analyses will consider patients with GOSE score of 7 and GOSE score of 8 separately. 4) Patient outcomes beyond 180 days post-TBI were not evaluated.

## Conclusions

We performed a clustering analysis and identified five sub-groups of mTBI based upon differences in patient socio-demographics, neurologic and psychiatric history, history of alcohol and tobacco use, receiving IV fluids in the ED, blood pressure, injury mechanism, and brain CT findings. Multi-dimensional patient outcomes differed amongst these sub-groups at 90 days and 180 days post-TBI. We plan to test the clustering structure identified in this study using a unique subject sample obtained from FITBIR and to use the knowledge gained from this study to build predictive models for TBI outcomes based upon data available at the time of initial evaluation of the patient with TBI.
